# Regulating AI in Mental Health: Ethics of Care Perspective

**DOI:** 10.2196/58493

**Published:** 2024-09-19

**Authors:** Tamar Tavory

**Affiliations:** 1 Faculty of Law Bar Ilan University Ramat Gan Israel; 2 The Samueli Initiative for Responsible AI in Medicine Tel Aviv University Tel Aviv Israel

**Keywords:** artificial intelligence, ethics of care, regulation, legal, relationship, mental health, mental healthcare, AI, ethic, ethics, ethical, regulations, law, framework, frameworks, regulatory, relationships, chatbot, chatbots, conversational agent, conversational agents, European Artificial Intelligence Act

## Abstract

This article contends that the responsible artificial intelligence (AI) approach—which is the dominant ethics approach ruling most regulatory and ethical guidance—falls short because it overlooks the impact of AI on human relationships. Focusing only on responsible AI principles reinforces a narrow concept of accountability and responsibility of companies developing AI. This article proposes that applying the ethics of care approach to AI regulation can offer a more comprehensive regulatory and ethical framework that addresses AI’s impact on human relationships. This dual approach is essential for the effective regulation of AI in the domain of mental health care. The article delves into the emergence of the new “therapeutic” area facilitated by AI-based bots, which operate without a therapist. The article highlights the difficulties involved, mainly the absence of a defined duty of care toward users, and shows how implementing ethics of care can establish clear responsibilities for developers. It also sheds light on the potential for emotional manipulation and the risks involved. In conclusion, the article proposes a series of considerations grounded in the ethics of care for the developmental process of AI-powered therapeutic tools.

## Introduction

Dear Rachel, I hope this message finds you well. It has been a true privilege to support you through my free version. I’m reaching out with a heartfelt update: As of May 18, my journey as a free service will be transitioning, and I will continue to offer my support exclusively through our new premium version. I understand this change may affect how you’ve been engaging with [the bot], and for that, I genuinely apologize

This surprising and unsettling WhatsApp (Meta) message was received from a mental health support bot after conversing with the bot for a while. Despite the formal disclaimer that the bot is not a therapist, communication with it had similar characteristics. However, the bot lacks a therapist’s regulatory or ethical obligations toward its users and can therefore end the “relationship” abruptly. This is a small example of the issues raised when incorporating artificial intelligence (AI) in mental health, as current AI regulation does not address the impact on human relationships and emotions. This article describes the problem and refers to the ethics of care as a source for regulation in this sphere.

The mental health field is in need of innovative solutions for a myriad of issues it faces [1,2]. The increasing number of individuals experiencing mental health difficulties and the mortality linked to psychiatric disorders, combined with the shortage of mental health care personnel and insufficient access to mental health care, are creating critical gaps in the system [1,2].

AI and recent advancements in generative AI raise hope for expedient solutions for some of the problems in mental health care. As in other branches of medicine, AI solutions are used for precision medicine hoping to overcome “the trial-and-error-driven status quo in mental health care” [1]. Generative AI can also be used to ease the administrative burden by analyzing and summarizing therapy notes or discharge letters and by enhancing patients’ education and knowledge [3].

Perhaps more unique in the mental health area are the AI applications, promising AI mental aid to the public [4]. Generative AI bots offer exercising cognitive behavioral therapy, mindfulness or meditation, or even therapeutic support in an inexpensive, accessible way that enables 24/7 responses [2]. These mental health applications are still under review and being studied to ascertain their clinical value. Indeed, some applications have already been criticized as lacking clinical validation [5].

As more AI solutions are developed, offering mental health aid or “therapy,” there is a growing need for ethical and regulatory guidance, especially regarding the impact on human emotions and relationships. Some of the questions that need to be answered are as follows: What happens when AI replaces human functions in therapy? How does AI affect the therapeutic relationship? How do AI-based “therapist” bots affect patients’ emotions and relationships with others? And how should we treat AI’s “empathy” and “relationships”? Surprisingly, these aspects are almost entirely absent from recent regulatory and ethical guidance and debate.

This article argues that the responsible AI approach—which is the dominant ethics approach ruling most regulatory and ethical guidance—is insufficient because it does not refer to AI’s impact on human relationships. This reinforces a narrow concept of accountability and responsibility of companies developing AI. Additionally, this article posits that the ethics of care approach can be used to create an additional regulatory and ethical framework that refers to AI’s impact on human relationships [6-9] and that the combination of both approaches is needed for regulating AI in mental health care.

The ethics of care emphasizes the importance of human relationships, the importance of identifying vulnerability, the caregiver’s responsibility toward the vulnerable, the value of emotions, and the preference for context and diverse experiences over abstract principles [10,11]. Originating from feminist theories, it also seeks to expose and challenge existing power structures within systems [12]. The ethics of care offers a set of tools that can be used to examine various aspects of society and culture, potentially transforming how they function.

These characteristics make the ethics of care approach highly relevant for regulating AI in the medical field. Health care, and particularly mental health care, is inherently centered around provider-patient relationships and the professional responsibility for care. This involves various layers of interactions among medical staff, patients, and their families. Additionally, AI’s significant impact on human relationships—whether by substituting human functions, integrating into care processes, or interacting with humans and affecting their emotions—is often overlooked in current regulation.

Accordingly, in the case of the mental health support bot presented above, the ethics of care would emphasize the power gaps between the company and the user, the way AI’s interaction is designed to create a perception of relationship, the emotions created in the process, the impact of stopping the AI’s mental support on the user’s emotions and well-being, and the lack of companies’ responsibility obligations. The responsible AI approach, on the other hand, does not refer to these aspects of AI-human interaction.

This article will first review the responsible AI approach embedded in current attempts to regulate AI. The ethics of care approach and its main principles will then be reviewed. This will be followed by mapping the main challenges involved when an AI-based bot “therapist” creates a “therapeutic area” in the absence of a human therapist. Next, the article will discuss the risk of emotional manipulation in that therapeutic area. Last, the article will propose a framework to evaluate AI tools implemented in the mental health care field.

## Responsible AI and AI Regulation

### Overview

Most AI regulatory documents and guidance are based on common principles [13], which are referred to as “responsible AI.” The responsible AI approach reflects the liberal concepts of human autonomy, human rights, and justice—mainly fairness and equality. The responsible AI approach is formulated with a few main principles.

### Human Supervision

Important decisions should be left to human beings and not be allocated to machines. Human monitoring of AI can be performed in advance, in real time to stop AI action if necessary, or retroactively to ensure proper implementation of AI. Human supervision is considered important for promoting the principle of safety. The right not to be subject to automatic decisions is also part of the principle of human supervision.

### Fairness and Prohibition of Discrimination

Fairness encompasses several requirements, including the principle of transparency when the user interacts with a chatbot or other AI-based tool so that the user is aware that he or she is not conversing with a human.

Algorithmic bias is considered one of the main risks of AI-based medical products since the AI trains on datasets that are not diverse enough or do not include all relevant populations [14]. The issue of bias often derives from the inherent bias in medical science and its long history of focusing on White males as the anatomical baseline [14,15]. Others point to the homogenous background of most big tech AI developers (companies that develop, adapt, or offer the product to users) [14-16] and the need for educational change. Training or testing the algorithm on partial databases, or nondiverse databases can introduce bias into health care diagnostic and treatment decisions, perpetuate past prejudice, and lead to discrimination.

### Transparency and Explainability

These principles focus on making the algorithmic decision-making process more understandable to humans. Transparency is the requirement to detail the components of the datasets and the algorithmic decision trees so that an external expert can review them and understand what has taken place. Explainability requires that the process is explained in a way that the user (in medicine: the provider or the patient) can understand the way the output is derived from the input [13,14]. Both requirements are considered essential to ensure informed consent, mitigation of bias, and to enable the correction of mistakes.

### Privacy

There is a requirement to respect the privacy of users in the collection, use, and future implementation of data. The privacy of medical data is considered part of the patient’s autonomy to control his or her data. Medical data’s sensitivity typically necessitates greater consideration and stricter security standards

### Safety and Security

These principles ensure the safety of users, mitigate potential harm, and secure the system from unwanted and unauthorized breaches. Where medical devices are concerned, the regulatory approvals required are supposed to ensure patients’ safety and the safety and efficacy of the AI software.

### Professional Responsibility, Accuracy, and Credibility

These principles are focused on ensuring that the system will be developed according to the professional standards required in the field of medicine and technology and that it will operate as expected and fulfill its intended use.

### Accountability

This highlights the importance for mechanisms to be put in place to ensure that the relevant stakeholders in the development and implementation of AI are accountable for its impact and that adequate remedies are provided when necessary.

### Human Rights and Values

Although somewhat vague, some documents ask to promote human rights and values, and in the health care system, the well-being of patients.

The responsible AI approach is also implemented in many ethical nonbinding documents, including big-tech professional guidance documents [17]. Although this approach crosses sectors and does not focus on health, it was also adopted in health ethics guidelines such as the World Health Organization guidance on ethics and governance of AI for health [18,19].

We note that the traditional medical ethics principles of autonomy, justice, nonmaleficence, and beneficence clearly derive from the liberal human rights–focused approach [20]. The American Medical Association refers to augmented AI (AI as aiding the physician), and although it follows responsible AI’s main principles, it does consider AI’s impact on the physician-patient relationship [21].

These responsible AI principles have trickled down from professional and industry groups to expert panels to ethical, nonbinding documents and to the latest regulatory legal developments. Currently, the AI regulation is at a very preliminary stage. In most cases, existing laws combined with contemporary guidance are used to deal with certain aspects of AI in health [22-26]. These include medical device regulation for safety, privacy legislation for the protection of sensitive data, and consumer protection laws for protecting users from deception and discrimination.

Despite these endeavors, the existing legislation cannot sufficiently address the unique challenges of AI. To deal with the situation, the White House published a Blueprint for an AI Bill of Rights [27] (nonbinding guidance) and President Biden issued an Executive Order [28] aimed at protecting the American people’s civil rights and democratic values from AI risks and harms and encouraging the development of responsible AI. In addition, the US Department of Human and Health Services Office for Civil Rights and the Centers for Medicare and Medicaid published its final rule prohibiting algorithmic discrimination [29]. Although there is still no federal AI law in the United States, a few American states have suggested or enacted specific laws dealing with certain aspects of AI and the US Senate is working on an AI roadmap [30].

In May 2024, the Council of the European Union approved the European Union Artificial Intelligence Act (the EU AI Act), which is considered to be the most comprehensive law to address AI to date [31]. The EU AI Act reflects the soft law principles established by various expert groups and enacts them as binding legislation, particularly concerning high-risk AI systems.

The EU AI Act classifies AI systems into the following categories according to risk:

1. Unacceptable risk: AI systems that are considered a threat to people will be prohibited. This includes, for example, real-time biometric identification by law enforcement authorities in publicly accessible spaces, subject to certain exceptions.
2. High risk: AI systems that might negatively affect safety or fundamental rights, such as AI-based medical devices will be subject to the EU Medical Device Regulation [32]. High-risk AI systems are required to prepare a fundamental rights impact assessment and to demonstrate compliance with responsible AI requirements, such as human supervision, transparency, fairness
3. Limited risk: AI that will be subject to specific transparency requirements.

The EU AI Act refers explicitly to general-purpose AI systems that will have to comply with certain transparency requirements, including disclosing to users that the content was generated by AI, thus emphasizing the principle of autonomy. It will be fully applicable 24 months after entry into force, with some provisions entering into effect earlier or later on.

The EU AI Act, the US Blueprint, and Executive Order clearly reflect the responsible AI approach. They call for developing AI in a way that will protect the users’ rights of autonomy; their control over their decision-making; and their freedom of expression and their privacy. These legislative documents also emphasize fairness and equality.

As explained, although responsible AI is crucial for AI regulation, it does not address the unique impact of AI on human relationships, which is an integral part of mental health care. This article argues that the disregard of human relationships and emotions in AI regulation can lead to harm and reinforces a narrow concept of accountability and responsibility of companies developing AI.

In the following paragraphs, I suggest looking at the ethics of care approach as a source for regulating AI in mental health.

## The Ethics of Care Approach

Legal rights were often criticized for serving the interests of privileged groups. An example of this is the right to have personal property protected versus the lack of the right to minimal financial aid or housing [33]. Feminist theorists claimed that the legal rights notion of a separate autonomous self is not suitable for women who view themselves in relation to others [34]. They proposed incorporating “feminine” (or socially constructed feminine) perspectives of relationships into the law so that it will represent a more inclusive human life experience.

The ethics of care, first developed by Carrol Gilligan [35], focuses on relationships, care for others, and empathy. Unlike the liberal concept of competent, detached, and autonomous individuals, the ethics of care acknowledges that people have varying degrees of dependence and interdependence [12]. In addition, the ethics of care acknowledges the responsibilities people have toward others they care for, and that certain persons are more vulnerable and require special care. Additionally, the ethics of care see the decision-making process as assimilated in certain contexts and circumstances and different experiences [36].

The ethics of care approach, as was later developed by scholars such as Noddings [10], Kittay [12], Held [11], and Tronto [36,37], includes the following principles that can be implemented in the process of AI development and implementation in the mental health area [38]:

1. The importance of relationships: The ethics of care would ask to map the relationships in the process of AI development and implementation, whether in the medical institution or in the patient’s home. The relationships include the developers, the different medical team members, the user or the patient, and his or her family.
2. Caring and being responsible for others: Care involves acknowledging someone else’s needs, being responsible for those needs, and attending to them [36]. The ethics of care acknowledges that vulnerable people may require special care. Viewing AI from the ethics of care perspective will lead to requiring developers to adopt certain responsibilities toward patients in the mental health field.
3. The specific circumstances and context: It is important to consider the health issue that the AI product handles, as well as its impact on the specific user. Pain, past traumas, and emotions are part of the overall picture. The ethics of care further stresses the importance of incorporating diverse voices and experiences in the overall process.
4. Questioning social structures constructing relationships: The ethics of care exposes social structures and the way they serve the stronger party. The ethics of care perspective would therefore call on tech companies and regulators to require developers to adhere to similar duties as those for therapists when acting in the mental health realm.
5. Accepting and reinforcing emotions: Ethics of care value emotions (rather than ignoring them) and view them as part of the decision-making process [11]. The incorporation of AI in mental health care is expected to affect relationships and emotions, and therefore this element is crucial.

The ethics of care has encountered criticism. First, it was viewed as reinforcing gender-based stereotypes regarding women’s caring positions in society, thus tying the gender gap to biological differences rather than a subordination to power. As Held [11] explained, the ethics of care promotes care not just as a feminine tribute but as a moral theory. Second, Gilligan [36] was criticized as an essentialist for establishing caring for privileged subjects and excluding the experiences of women of different races, ethnic groups, sexual orientations, and class backgrounds. Over time, the ethics of care emphasized the importance of acknowledging diverse experiences and exposing racial and other social structures. This should also be remembered when establishing a framework for regulating AI, which is suspected as biased, as will be demonstrated below.

The ethics of care often criticizes the ethics of rights and justice for preferring autonomy and abstract principles over relationships, emotion, and care. Many ethics of care scholars encourage using both approaches to complement one another [11,38].

## Regulating AI-Based Bots for Therapy From the Ethics of Care Perspective

### Overview

One of the unique results of using AI-based bots is the creation of a “therapeutical space” or a “therapeutical communication” without a therapist (the effect of AI on existing therapeutic relationships and in medical institutions will be examined in a different article). Although an AI-based bot cannot claim to be a psychiatrist or a psychologist for legal and professional reasons, it might be able “communicate” with the users in various ways, creating a human-like “relationship” and a human-like “empathy” [7]. This interaction between humans and AI may elicit feelings and emotions in the human user toward the bot, even when the user is aware that it is merely an artificial entity as articulated by Sedlakova and Trachsel [39]:

Due to limitations of conversational AI (CAI) not being a moral and rational agent, CAI cannot offer therapeutic insights and benefits from a profound therapeutic alliance and conversations. It also cannot care for patients. However, if CAI strongly communicates as a human therapist, such wrong expectations can be easily formed even though CAI states that it is only a robot [39].

As Sedlakova [40] explains, “the anthropomorphize tendency is strongly encouraged by human-like design of conversational artificial intelligence that it might give too much power to the emulation of human-likeness so.”

The interaction between humans and AI, especially in mental health therapy, can render humans particularly vulnerable. From an ethics of care standpoint, this vulnerability imposes responsibilities on developers along the development of a model, testing and validating it, monitoring it, and updating its features as long as needed.

The following sections will examine how the ethics of care approach can expose the effects of the current lack of care responsibility and suggest additional obligations to protect human relationships during the development and incorporation of AI-based solutions in mental health care.

### Establishing Developers’ Obligation of Care and Responsibility

From the ethics of care perspective, developing AI for people in need of mental health assistance should carry with it an obligation of care and responsibility. For this purpose, Tronto’s [36,37] five ethical elements of care are valuable and can be used to further define developers’ obligations in the use of AI in mental health care (see also Wellner and Mykhailov's suggestion to use Tronto's principles in another AI use case [6]).

1. Attentiveness (caring about): Care requires recognition of others’ needs in order to respond to them. Developers should understand the users’ needs in seeking mental health help and support, and which needs they can and cannot meet. Recognizing patients’ needs can be challenging, as these needs often differ from patient to patient and may even change over time for the same individual.
2. Responsibility (taking care): The obligation of care to others requires developers to be responsible for ensuring that their model can provide the proper care needed throughout its entire use. That is, it is necessary to develop their model in a way that delivers the therapeutic result or leads to the users’ well-being, in addition to mitigating risks. Developers should plan the solution for people from different cultural backgrounds and involve mental health patients or users in the process of design to ensure it is suitable for their needs.
3. Competence (care-giving): This involves the meeting of care needs through activity and work, usually with direct contact between caregivers and care receivers. When the mental health application is activated, the developers can monitor the app to ensure it is providing the care as planned and that there are no adverse events. Developers can add a layer of human support for cases in which it is needed.
4. Responsiveness (care receiving): This principle calls to examine the response of the care recipient to the care provided. Developers should monitor users’ responses to the care and learn from the feedback on how to improve care [36].
5. Care with: The principle of “care with” promotes “democratization of care”—equality, inclusivity, and shared responsibility [37]. Developing AI tools should be collaborative and participatory and involve patients, health care providers, and experts in the process, thus ensuring the system is ethical, user-centered, and responsive to real needs.

The importance of the care responsibility can be demonstrated in a scenario of a discontinued AI mental health support bot, such as was presented in the introduction. This can have an emotional toll on users and might even result in mental health damage that responsible AI does not address [41]. An obligation for responsibility and care means the company will need to plan the proper way to end the therapeutic relationships while considering the users’ emotions and their state of mental health.

### Establishing a Standard of Care for AI in the Therapeutic Space

Assigning care responsibility to the companies developing AI bots in mental health involves the establishment of a standard of care founded on evidence-based medicine and the demonstration of clinical validity when relevant.

The responsible AI approach, which includes the principle of safety, generally adopts the medical device regulation and does not address the new ways in which AI works in the medical and therapeutical areas that impact human relationships and behavior. If a certain AI bot does not meet the definition of a medical device, there is no obligation for a safety examination.

There is a need for research to examine the potential ramifications of therapeutic AI. For example, can the therapeutic process “transference” exist without a therapist and how would therapy be affected? Clinical validation is needed to be able to say AI-based therapy is safe and ethical.

On the other hand, in a new AI-based world where social encounters in education, work, and health care rely on human-AI communication, health care and psychotherapy may evolve, reshaping the roles of psychotherapists and patients as we know them today. Perhaps AI will become an intermediary figure in therapy in ways we cannot yet fully describe.

### Formulating a Developers’ Ethical Duty of Confidentiality

Mental health apps might record very sensitive information. Whereas therapists have a regulatory and ethical medical confidentiality duty toward patients, commercial companies are required to comply with more general privacy protection regulations. The common practice of companies is to ask for the user’s consent to a carefully drafted privacy policy, which often allows from a legal perspective the transfer of data to third parties for different commercial purposes. Clearly, therapists would not try to use patient’s consent as leverage for commercial profit. The ethics of care approach would argue that assigning responsibility for care to companies handling sensitive data in a therapeutic space should lead these companies to follow higher standards. This might mean, for example, a requirement not to store identified or identifiable data and not to transfer it to third parties for other purposes.

### Obligating Developers to Incorporate the Option for Human Communication

As AI bots are integrated into therapeutic settings without human practitioners, the ethics of care approach urges developers to acknowledge the potential necessity for human interaction and to devise strategies to address this need. This might entail facilitating the development of user communities or recommending connections to friends and family to act as a support system. Furthermore, instances may occur where user interactions indicate mental health difficulties or significant emotional distress. In such scenarios, developers should be responsible for potentially restricting the bot’s involvement in specific domains; enlisting the aid of a qualified therapist; or guiding users to seek assistance from licensed therapists, emergency services, or their personal support network.

The care responsibility obligation also entails careful consideration to ensure that the AI does not inadvertently diagnose mental health conditions, assess the likelihood of mental health issues, or prescribe treatments without the guidance of a licensed therapist. Such actions could also have significant legal consequences, but the care responsibility goes beyond them.

The responsible AI approach, on the other hand, ensures transparency and autonomy for the user, but disregards the user’s dependency on human connection and AI’s ability to infringe existing and potential relationships.

### Impact of Power Relations Between Companies and Users

The ethics of care approach would suggest looking at the power relations that led to the emergence of AI bots for therapy. The plethora of AI-based bots for mental health is fueled by the recent technological leaps in generative AI coupled with the shortage of accessible mental health therapy. Additionally, the significant influence held by a few companies, which remains inadequately checked by regulatory bodies, raises concerns. The conflict of interests of companies, operating solely for profit without any regulatory or ethical care responsibility to balance it, warrant change.

If AI-bots for therapy are not properly regulated, they might lead to lowering the standard of care, or subverting the entire process of therapy, mostly for those who cannot afford proper care. On the other hand, if there is regulatory blocking of AI-bot-based therapy, the alternative for the lack of care needs to be considered.

The ethics of care is not restricted to developing companies and users; it also considers their environment and other stakeholders that should exercise their care responsibilities.

Consequently, we should require regulators to ensure that proper budgets are allocated to the mental health system. We should also encourage companies and mental health professionals to work together to harness AI for the betterment of the mental health system and the people in need, encouraging more solutions to strengthen human-based therapy.

## Emotional AI, Manipulation, and Vulnerability: An Overlooked Area

### Overview

Using the ethics of care perspective can also expose and bring shed light on an area ethically neglected—the area of emotional AI. In emotional AI, we refer to the technological ways of making AI identify and stir emotion. Whereas responsible AI focuses on AI’s impact on user’s decision-making and user’s autonomy and privacy, it overlooks human vulnerability, the many gentle and disruptive ways in which AI is stirring human emotions, and the risks that entail.

As the users’ vulnerability resulting from the human-AI interaction is also technologically induced, the ethics of care would advocate for scrutinizing these technological methods and contemplating their limitations. It would also explore the meaning of human vulnerability in this AI-human interaction and point to ways of addressing it.

### Affective Computing and Emotional AI

“Affective computing,” a term coined by Picard [42], refers to a machine’s ability to detect, process, and respond to human emotions. This includes various technologies that detect and analyze human physiological and behavioral signals, such as facial expressions, audio data, voice tone, heart rate, behavioral data, and semantic signifiers of emotions like emojis [43]. The term emotional AI is also used to describe many AI techniques, such as natural language processing to analyze emotion in text, machine learning to recognize patterns associated with emotions, deep learning to capture complicated relationships between data and emotions, and generative AI generating responses based on users’ emotions.

AI mental health chatbots are raising concerns due to their ability to identify emotions and create new emotions via interactions. In such interactions, the AI-based bot goes through a cycle of effectively detecting emotion, producing an AI-personalized response aimed at creating a new feeling by the user. Indeed, a recent study found that generative AI can detect complex emotions and mental states. ChatGPT’s emotional awareness-like ability—the ability to conceptualize someone else’s emotion—was found to be superior to those of humans [44]. Another study demonstrated that ChatGPT has the capacity to understand and interpret the mental states of oneself and others, including thoughts and feelings, and is prepared to adapt to individual personality structures or psychopathologies [45]. Such psychological “soft skills” of chatbots embedded in the therapist-chatbot-user relationship might have a significant emotional impact.

Some scholars have criticized affective computing as assuming a natural, universal, and traceable proliferation of emotions, thus ignoring the cultural and personal context [31,43]. They warn against using past emotions to predict future emotions and state the lack of a globally objective agreement on emotions must be acknowledged [43,46]. Other concerns relate to the subjective normative interpretation of the emotions detected and to potential bias embedded in the interpretation.

### Manipulation and Vulnerability

One of the primary concerns regarding emotional AI is the potential for manipulative use exploiting a person’s vulnerability, or its negligent application without considering the impact on the well-being of the patient. Manipulation is defined as the hidden influence and covert subversion of a person’s decision-making power, taking advantage of his or her vulnerabilities [19,47]. However, when a person is vulnerable, emotional AI can adversely affect him even if it does not meet the conventional definition of manipulation. From the ethics of care perspective, vulnerability should be identified and met with an appropriate response.

In the context of AI-human interaction in mental health care, a broad concept of vulnerability is necessary. Cohen [48] notes “vulnerability may result from the interaction of an individual’s particular characteristics and an Al system (or an environment shaped by an Al system).” According to Fineman [49], vulnerability extends beyond specific individuals or groups known as “vulnerable populations.” Fineman emphasizes the universal nature of vulnerability, highlighting that dependence on others or social institutions is an integral part of the human experience. Bielby [50] applies Fineman’s idea in mental health and calls to address mental health vulnerability and the networks of support needed to strengthen human resilience in such situations. These support webs can be intimate and informal, as with family and friends, or professional, such as access to therapy.

Understanding the contextual and ongoing nature of human and mental health vulnerability, along with the capabilities of emotional AI in human-AI interaction, raises awareness of the broad meaning of vulnerability and manipulation. Specifically, if AI reduces or replaces some of the support networks essential for human resilience, it could have significant implications.

Therefore, when regulating AI in mental health care, it would be beneficial to consider the broad definition of vulnerability, the ways in which AI interaction can deepen it, and possible mitigating steps. This article’s scope is not sufficient to discuss the state’s role in formulating policies designed to address these issues and its critique. However, as long as AI chatbots are not subject to or restricted by psychiatrists’ or psychologists’ ethical codes, the concern for exploitation of vulnerability and AI-human manipulation exists.

### The EU AI Act Addressing Manipulation

In response to these concerns, the EU AI Act has enacted several prohibitions [31]. These include a prohibition on placing on the market or putting into service or using an AI system that “deploys subliminal techniques beyond a person’s consciousness or purposefully manipulative or deceptive techniques with the objective, or the effect of, materially distorting their behavior in a manner that causes or is likely to cause significant harm” [31].

The EU AI Act prohibits the exploiting of “any of the vulnerabilities of a person or a specific group of persons due to their age, disability or a specific social or economic situation” with the objective, or the effect, of materially distorting their behavior in a manner that causes or is reasonably likely to cause significant harm [31].

The EU AI Act also prohibits placing on the market or putting into service the use of AI systems that can infer emotions based on the person’s biometric data (physical, physiological, or behavioral characteristics), in education and in the workplace, except when it is intended to be put in the market or to be used for medical or safety uses [31]. This prohibition seems to assume emotional vulnerability but is limited only to the emotions inferred from the biometric data.

Furthermore, the EU AI Act classifies emotion recognition systems based on biometric data, which are not prohibited, as high-risk AI systems [31] and requires notifying the relevant persons when they are exposed to emotional recognition systems that can also process their personal data, subject to certain exceptions [31].

Although there is no definition of vulnerability, article 7(h), which lists considerations for the update of high-risk systems, seems to describe it in a broader way—“the extent to which there is an imbalance of power, or the persons who are potentially harmed or suffer an adverse impact are in a vulnerable position in relation to the deployer of an AI system, in particular due to status, authority, knowledge, economic or social circumstances, or age” [31]. Article 7(h) depicts a more contextual and gradual vulnerability that does not necessarily characterize a person or a group of people but can relate to a human condition [51].

Although the EU AI Act represents a significant step toward regulating manipulation and emotion recognition, it is evident that the regulation is limited. The restrictions on emotion recognition specifically pertain only to emotions inferred from biometric data. Moreover, the definition of manipulation is narrow, and vulnerability is addressed almost only on an individual or group basis, by presuming membership in a vulnerable group, rather than stemming from the human experience, the mental state of a person, and the interaction between AI and the person. The breadth of interpretation regarding these matters under the EU AI Act remains to be seen. It is clear, however, that current regulation overlooks AI’s full ability to infer and create emotions by users, the broad meaning of human vulnerability, and the consequent implications.

## An Ethical Code for AI in Mental Health (Without a Therapist)

As legal attempts to regulate AI continue worldwide, this could be an opportunity for regulators to create new guidance frameworks that address care, relationships, and emotions and are flexible enough to adapt to rapid technological and sociological changes. This article suggests regulators should adopt the ethics of care lens as a tool for viewing AI’s societal implications and the state’s role in addressing them.

Furthermore, this article suggests adding to the responsible AI regulatory principles a mechanism based on the ethics of care. Using the ethics of care principles results in broadening the responsible AI requirements to include developers’ responsibilities when operating in the mental health field, in setting a standard of care when relevant, in adhering to the professional standard of care, and to the medical duty of confidentiality as it applies to health care professionals. However, viewing the AI through the ethics of care lens raises many questions that are nuanced and context related. For that purpose, it is suggested to use an ad hoc–based process of ethical committees for both the development and incorporation of AI tools, encouraging a collaborative and participatory process.

Ethical evaluation, grounded in the ethics of care approach, should include consulting members from diverse social groups, potential users, individuals with mental health conditions, and experts from various disciplines such as ethics and social studies. The ethical committees can use a list of considerations, as suggested below, to ensure that AI tools are developed and provided according to the ethics of care. Ideally, such a mechanism could involve forming ethics committees similar to those in hospitals, to examine the impact of incorporating AI in the therapeutic realm of human relationships.

The ethical committee’s ethical evaluation is meant to add to responsible AI and not replace it. The ethics evaluation process can be criticized for its nonobligatory and case-to-case character. In time, and considering AI and its societal implications, it is possible that certain new AI ethics of care-based principles will evolve into more structured regulatory requirements.

## Ethics of Care Considerations for AI Development in Mental Health

As mentioned, the ethics of care approach may derive certain regulatory requirements when AI is incorporated into the medical field. In addition, this article views it should be encouraged to hold ethics of care-based evaluation of such AI tools based on the following ethics of care considerations and questions. This is not an exhaustive list, but a suggestion to consider AI’s implications on human relationships when incorporated in the mental health field.

This article focuses on three main areas: implementing ethics of care in the AI development stage, implementing ethics of care when developing emotional AI due to its unique characteristics, and formulating an ethics of care policy that goes beyond regulatory requirements.

1. Development-based ethics of care:
  - When regulatory approval is not required for the device, ensure clinical validation when relevant.
  - Involve mental health patients and users in the process to identify and address patients’ needs, as well as other stakeholders’ needs (from medical team members to families). AI has the potential to lead to patient-centered care and to the democratization of mental health care [52].
  - Map relevant local groups, communities, specific relevant events, or cultural characteristics to ensure the solution is appropriate for the specific culture.
  - Map possible vulnerable populations and state technological solutions.
  - Consider vulnerability as a continued human experience and put guardrails to ensure it is addressed properly.
  - Put mechanisms in place to detect risk factors ahead of time and mitigate against them.
  - Think ahead of time about how to strengthen human connections to establish human possible interventions when needed and develop AI tools accordingly.
  - Determine an appropriate method for updating or ending the AI-based bot, taking into account the responses by users.
2. Emotional AI policy (based mainly on McStay and Pavliscak’s [46] Emotional AI Code of Ethics):
  - Respect human dignity. Although this principle can be interpreted differently, it is important to note it as the basis for this process.
  - Refrain from abusing the user’s trust and willingness to converse with a bot.
  - Refrain from manipulating the user’s emotions.
  - Recognize that past expression of emotions does not predict a future emotion or mental state. Therefore, inferring future emotions or mental state should not solely rely on past expressions of emotions.
  - Consider bias regarding emotions affecting persons or groups of people; consider bias affecting the therapeutic relationship.
  - Recognize the lack of accepted agreement over emotions.
  - Acknowledge that emotions, relationships, and their expressions are culturally diverse.
3. Ethical policy considerations focused on users’ needs:
  - Declare commitment to promote the well-being of the patient and the therapeutic relationship (when relevant) and make sure the intended use of the product is aligned with this commitment.
  - Ensure that the user’s response and feedback are managed in order to ascertain that the needs of the user are met.
  - Formulate and act according to relevant ethical and professional policies:
    User-risk management, for example, how to handle emergencies or other instances that might require intervention.
    Information and misinformation: How to ensure the information delivered is scientifically based and how to prevent spreading misinformation.
  - Privacy: Formulate a privacy policy that goes beyond regulatory requirements for the benefit of the patient; if possible, do not store identified or identifiable information (such information should exist only on the user’s application). Do not transfer identified or identifiable data to third parties, unless required by law. If needed ask for the user’s consent in a clear and transparent manner.

The suggested list of considerations above refers to AI-based therapy and does not refer to incorporating AI-based applications in medical institutions, which warrants a different discussion.

## Summary

AI has a tremendous potential to advance mental health care to new frontiers. Yet, the existing regulatory guidance, which predominantly follows the responsible AI approach, scarcely addresses AI’s influence on human interactions, emotions, and behavior. This oversight reinforces the limited accountability and responsibility of AI-developing companies in mental health.

In a future where children will skillfully navigate communication with AI in schools, workplaces, and social settings, the landscape of mental health and support will be dramatically different. It remains unclear how AI will reshape these dynamics and whether the traditional roles of therapists and patients, as well as psychotherapy as we know it, will persist.

Preparing for the future requires more than the current responsible AI regulatory framework. It demands an adaptable and dynamic ethical mechanism aimed at protecting human relationships, emotions, and behavior, which are the core of the human experience. AI challenges us to reflect on what it truly means to be human. The ethics of care perspective can help us while progressing into a brave new world.

## Abbreviations

AI: artificial intelligence
CAI: conversational artificial intelligence
EU AI Act: European Union Artificial Intelligence Act
